# Potential Confounders of the Different Impact of Alcohol Intake Between Sexes on the Incidence of Atrial Fibrillation

**DOI:** 10.1002/joa3.70201

**Published:** 2025-09-21

**Authors:** Yu Nomoto, Naoya Kataoka, Teruhiko Imamura

**Affiliations:** ^1^ Second Department of Internal Medicine University of Toyama Toyama Japan

**Keywords:** catheter ablation, metabolic syndrome, women

To editor,

The association between alcohol intake and the development of atrial fibrillation (AF) has been well documented across various populations. Nevertheless, the potential influence of sex differences on this relationship has remained unresolved. The present study provides important insights by showing that higher alcohol intake was consistently associated with an incremental risk of AF in both sexes, while even lower levels of alcohol intake conferred an elevated risk only in men, but not in women [1]. These findings are intriguing and merit careful interpretation.

The underlying pathophysiological mechanisms responsible for the observed sex‐specific differences are still uncertain. The authors suggested that different beverage preferences between sexes may play a role [1]. Women may consume wine more frequently than men, and wine contains potentially protective compounds such as polyphenols. However, this explanation remains insufficient because prior epidemiological studies have also demonstrated that heavy wine consumption in women is associated with a higher incidence of AF.

The interaction between alcohol consumption and dietary patterns should not be overlooked. Alcohol is commonly consumed alongside meals, and certain dietary components may influence the risk of AF. For example, the Mediterranean diet, which is generally considered cardioprotective, includes abundant marine omega‐3 fatty acids, yet recent evidence paradoxically suggests that high omega‐3 intake may increase the risk of AF [2]. Such factors could act as hidden confounders when interpreting the relationship between alcohol and AF prevalence.

Lifestyle‐related factors that differ by sex may have contributed to the findings. Recent studies have linked low‐carbohydrate diets, sometimes preferred by Japanese women, with an increased incidence of AF [3]. Moreover, differences in body composition, hormonal milieu, alcohol metabolism, and the prevalence of comorbidities such as hypertension or obesity may all interact with alcohol exposure to influence AF risk differently in men and women [4]. These factors highlight the complexity of disentangling the causal pathway linking alcohol to AF across sexes.

While the cross‐sectional nature of the present study provides valuable epidemiological evidence, it cannot establish causality. Well‐designed prospective cohort studies, and ideally randomized controlled trials stratified by sex, will be required to confirm whether alcohol restriction can effectively reduce the incidence of AF.

## Ethics Statement

The authors have nothing to report.

## Consent

The authors have nothing to report.

## Conflicts of Interest

The authors declare no conflicts of interest.

## Data Availability

The manuscript does not include any original data.
